# Three-Dimensional Shape and Deformation Measurements Based on Fringe Projection Profilometry and Fluorescent Digital Image Correlation via a 3 Charge Coupled Device Camera

**DOI:** 10.3390/s23156663

**Published:** 2023-07-25

**Authors:** Wei Sun, Zhongda Xu, Xin Li, Zhenning Chen, Xinqiao Tang

**Affiliations:** College of Aerospace Engineering, Nanjing University of Aeronautics and Astronautics, Nanjing 210016, China

**Keywords:** 3CCD color camera, fluorescent speckles, fringe projection profilometry, telecentric lens, digital image correlation

## Abstract

We propose a novel hybrid FPP-DIC technique to measure an object’s shape and deformation in 3D simultaneously by using a single 3CCD color camera, which captures the blue fringe patterns and red fluorescent speckles within the same image. Firstly, red fluorescent speckles were painted on the surface of the specimen. Subsequently, 12 computer-generated blue fringe patterns with a black background were projected onto the surface of the specimen using a DLP projector. Finally, both the reference and deformed images with three different frequencies and four shifted phases were captured using a 3CCD camera. This technique employed a three-chip configuration in which red–green–blue chips were discretely integrated in the 3CCD color camera sensor, rendering independent capture of RGB information possible. Measurement of out-of-plane displacement was carried out through the implementation of Fringe Projection Profilometry (FPP), whereas the in-plane displacement was evaluated using a 2D Digital Image Correlation (DIC) method by leveraging a telecentric-lens-based optical system. In comparison to the traditional FPP-DIC hybrid methodology, the present approach showed a lower incidence of crosstalk between the fringe patterns and speckle patterns while also offering a corrective for the coupling of the in-plane displacement and out-of-plane displacement. Experimental results for the in-plane cantilever beam and out-of-plane disk comparisons with the traditional 3D-DIC method indicated that the maximum discrepancy obtained between FPP-DIC and 3D-DIC was 0.7 μm and 0.034 mm with different magnifications, respectively, validating the effectiveness and precision of the novel proposed FPP-DIC method.

## 1. Introduction

Digital Image Correlation (DIC) [1], as a non-contact optical measurement technique that provides full-field measurements, has become a popular tool in the realm of experimental mechanics for quantifying deformation. Given its versatility, relatively low environmental demands, and high measurement precision, the DIC method has gained widespread usage in multiple domains, including materials science, biology, aerospace engineering, microelectronics, and so on [2,3,4,5]. The fluorescent DIC method was originally introduced by Berfield [6] as a means of achieving nano-scale deformation measurements. In contrast to ordinary speckles, fluorescent speckles [7,8,9] possess several unique advantages, including exceptional contrast, elimination of specular reflectance, and the ability to measure multiple surfaces simultaneously. It is important to note that fluorescent speckles are invisible under white light illumination, necessitating their illumination with ultraviolet (UV) lamps of specific wavelengths, which then allows for the capture of light intensity by the camera sensor. In recent years, the distinctive qualities of fluorescent speckles have led to their extensive utilization in the assessment of the deformation response of thin films, composite materials, cells, batteries, and other materials [10,11,12].

However, 2D-DIC operations are frequently used in material science and mechanics for detecting and tracking in-plane displacements and strains. Though easy to operate and requiring only a single camera, 2D-DIC methods are limited in their ability to measure deformations in three dimensions. In contrast, 3D-DIC methods can offer highly precise measurements of 3D deformations, but their implementation demands synchronized triggering of multiple cameras, thereby increasing operational complexity. Moreover, DIC techniques that employ subset-based correlation operations may encounter challenges in measuring the 3D shape of complex objects accurately.

Concurrently, Fringe Projection Profilometry (FPP) [13,14,15,16] has gained popularity as a preferred method for 3D shape measurement. By employing the procedures of phase shifting [17,18,19], phase unwrapping [20,21,22,23,24], and phase–height mapping [25,26,27], the 3D point cloud may be effectively reconstructed for the relevant object. However, the FPP method relies on projector-based fringe projections and can achieve high-precision 3D reconstructions by resolving the object phase. However, owing to the nature of the projected images, FPP methods are not well suited for measuring deformation.

By expertly integrating DIC and FPP methods, it is possible to overcome their respective limitations and enhance the overall accuracy and coverage of deformation measurements. Tay [28] and other researchers employed Fourier Transform-based methods to determine out-of-plane displacement and implemented DIC to detect in-plane displacement. Nonetheless, the effectiveness of frequency domain filtering was limited in filtering the fringe patterns. Furthermore, the two techniques were implemented separately in the aforementioned study, and the interdependence between the in-plane and out-of-plane displacements was neglected, which restricted the ability to measure 3D rigid body displacement exclusively. Incorporating the four-step phase-shifting technique with DIC, Shi Hongjian [29] and co-researchers successfully investigated the profile and tensile strain of plastic sheets. The proposed method in this study demonstrated the efficacy of the four-step phase-shifting patterns that effectively eliminate the fringe pattern. However, it is worth noting that the current study did not take into account the potential coupling effects between the in-plane and out-of-plane displacements and neglected to modify the outcomes of the 2D-DIC analysis. Luis et al. [30] employed red pigment to generate speckles on the test piece and projected blue fringe patterns onto its surface. A single CMOS color camera was subsequently utilized to segregate the speckle pattern and the fringe pattern. Despite this, discernible color crosstalk persisted in the segregated patterns.

To address this issue, the present study employed a numerical method to rectify the in-plane displacement. However, with regard to calculating the out-of-plane displacement, a subtraction of the phase before and after deformation was simply carried out. Therefore, if there were any in-plane displacements, the pixel coordinates between the reference plane and the deformed plane would vary, rendering direct subtraction an unsound approach. Subsequently, the technique was employed by Luis [31] in a vibration test. To circumvent stroboscopic issues, Luis [32] and colleagues proceeded to substitute projection fringes with laser fringes. Notably, Wu Zhoujie [33] and associates employed three-step phase shifting and gray code technology to infer the 3D shape of an object, subsequently matching it via DIC. The direct subtraction of the 3D coordinates of the corresponding point shape enabled the determination of the 3D displacements. This approach significantly broadened the scope of the FPP-DIC hybrid method, thereby uncovering its potential for measuring arbitrary object morphologies. Unfortunately, the practicality of using median filtering to remove speckles depends on the size and color depth of the speckles, which makes it ineffective in certain situations. Zhang [34] and others proposed a fringe image extraction method based on deep learning technology that transformed speckle-embedded fringe images into speckle-free fringe images. However, deep learning methods require training a large number of samples and may not be able to cope with complex surface conditions of objects.

The popular FPP-DIC hybrid method not only offers an economical, streamlined, and space-efficient substitute for the conventional 3D-DIC method but also provides the capability to concurrently determine the 3D shape and deformation of intricate objects. It is evident that this method serves as a significant complement within the domain of 3D shape and deformation measurements while additionally manifesting tremendous potential for a wide spectrum of practical applications [35,36].

The present study combined the FPP method and the DIC method with the innovative incorporation of fluorescence speckle technology, a 3CCD color camera, and a telecentric lens. Compared with the FPP-DIC method mentioned above, the greatest innovation of this article was the use of color to separate speckle patterns and fringe patterns. This method does not require consideration of factors such as speckle mode and complexity of the measured object’s shape, making it applicable to any situation. This minimizes the crosstalk between speckle and fringe patterns and achieves high-precision 3D measurement of shape and deformation. In addition, we used fluorescent speckle instead of ordinary diffused speckle, which increased the accuracy of the DIC operation. We also used a telecentric lens to eliminate the coupling between the in-plane displacement and out-of-plane displacement. Experimental evaluations, including the rigid body translation experiment, disk center-loading experiment, and cantilever beam free-end-loading experiment, revealed that our novel FPP-DIC hybrid approach yields accurate and reliable results, thereby fulfilling the practical requirements of engineering measurements.

## 2. Principle

### 2.1. Separation of Fringe Patterns and Speckle Patterns

**Figure 1:**
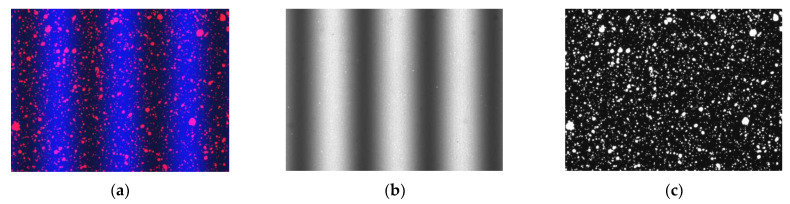
Separation of fringe pattern and speckle pattern. (**a**) Color image, (**b**) Blue channel image, (**c**) Red channel image.

**Figure 2:**
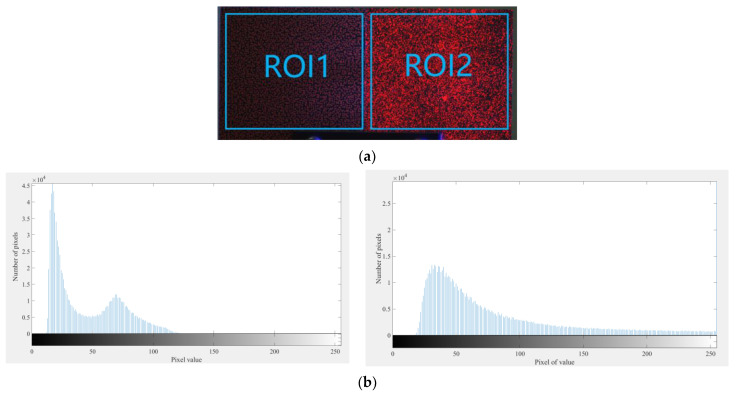
Comparison of diffuse reflection speckles and fluorescent speckles. (**a**) Diffused reflection speckles (ROI1) and fluorescent speckles (ROI2), (**b**) Histograms of ROI1 and ROI2.

Figure 1a presents a 24-bit RGB color image obtained with a 3CCD color camera that comprises a blue fringe pattern and a red fluorescent speckle pattern. In order to isolate the patterns, the three channels of the image were extracted. Subsequently, the extracted blue and red channel images were transformed into 8-bit grayscale representations, as demonstrated in Figure 1b,c, respectively. Notably, the proposed method effectively separated the fringe pattern and the speckle pattern with minimal crosstalk between the two patterns. It should be noted that we used fluorescent speckles instead of normal diffuse reflection speckles to reduce the aperture of the camera lens to ensure that the captured image did not experience overexposure. At this point, if a normal diffuse reflection red speckle pattern was used, the image in the red channel would be low in brightness, resulting in poor speckle pattern quality and thereby affecting the accuracy of the DIC calculation. The fluorescent speckle pattern could achieve spontaneous luminescence through UV lamp irradiation, greatly enhancing the quality of the speckle pattern and significantly improving the accuracy of the DIC calculations. Figure 2a shows a comparison between the diffused speckles (ROI 1) and fluorescent speckles (ROI 2) under the condition of a small aperture. The histogram in Figure 2b reveals that the grayscale of diffused speckle was mainly concentrated between 0 and 100, resulting in low image brightness. On the other hand, the grayscale distribution of the fluorescent speckles was more uniform, leading to higher-quality speckle patterns.

### 2.2. Calculation of Out-of-Plane Displacement Using FPP

The FPP method can measure a 3D shape by projecting fringes onto the surface of the object and performing phase recovery and phase unwrapping. The expression for fringe pattern is shown below:

(1) $$I_{i}\left(x,y\right)=I\prime \left(x,y\right)+I\prime \prime \left(x,y\right)\cos\left[\varphi \left(x,y\right)+\delta_{i}\right]$$

where $I_{i}\left(x,y\right)$ is the intensity distribution of the fringe pattern; $I\prime \left(x,y\right)$ is the average grayscale value of the image; $I\prime \prime \left(x,y\right)$ is the modulated grayscale value of the image; $\varphi \left(x,y\right)$ is the phase to be calculated; and $\delta_{i}$ is the phase-shifting value of the image. This article used the four-step phase-shifting algorithm, where $\delta_{i}$ is 0, 90°, 180°, and 270°, respectively. The calculated phase is:

(2) $$\varphi \left(x,y\right)=\arctan\left(\frac{I_{4}-I_{2}}{I_{1}-I_{3}}\right)$$

The three-frequency four-step algorithm can unwrap the phase $\varphi \left(x,y\right)$ into the absolute phase ϕ. The fringe frequencies used in this article were 70, 64, and 59. These three frequency fringes can be synthesized into a fringe with a frequency of 1 by combining them in pairs, thereby completing the phase unwrapping.

The phase–height method employed in FPP serves to determine the out-of-plane displacement:

(3) $$\left\{\begin{array}{l} w=k\Delta \phi \\ \Delta \phi \left(x,y\right)=\phi_{2}\left(x+u,y+v\right)-\phi_{1}\left(x,y\right) \end{array}\right.$$

The displacement *w* occurring out of plane can be computed using the formula presented in this study. A system constant *k* is employed that may be obtained by means of a linear fitting approach utilizing a simple rigid body translation; additionally, the phase difference Δϕ required for the computation can be determined via the application of the phase-shifting method and a time-unwrapping algorithm. The in-plane displacement $\left(u,v\right)$ was determined using 2D-DIC method, and the image coordinate $\left(x,y\right)$ was also taken into account. It was noteworthy that, in accordance with the presented Formula (1), the subtraction of the phase value was not direct; rather, the corresponding phase value for the identical coordinates prior to and following the deformation event was subtracted. This approach took into consideration the effect of in-plane displacement on out-of-plane displacement, resulting in a more rigorous methodology than a simple phase-difference technique.

### 2.3. Calculation of in-Plane Displacement Using 2D-DIC Based on a Telecentric Lens

The 2D-DIC algorithm, which relies on image gray value, offers a means of matching that is capable of tracking the motion of subsets via correlation operations. By leveraging this approach, it is possible to derive both full-field displacement and strain measurements for the object being analyzed. Among the various correlation functions that have been explored, the Zero-Normalized Sum of Squared Differences (ZNSSD) has garnered particular attention due to its stability and robustness:

(4) $$C\left(\vec{p}\right)={\sum_{i=-M}^{M}\sum_{j=-N}^{N}\left[\frac{f\left(x_{i},y_{j}\right)-f_{m}}{\sqrt{\sum_{i=-M}^{M}\sum_{j=-N}^{N}{\left(f\left(x_{i},y_{j}\right)-f_{m}\right)}^{2}}}-\frac{g\left(x_{i}^{\prime },y_{j}^{\prime }\right)-g_{m}}{\sqrt{\sum_{i=-M}^{M}\sum_{j=-N}^{N}{\left(g\left(x_{i}^{\prime },y_{j}^{\prime }\right)-g_{m}\right)}^{2}}}\right]}^{2}$$

where *M* and *N* represent the length and width of the subset; $f\left(x,y\right)$ and $g\left(x\prime ,y\prime \right)$ represent the gray distribution of the subset of reference image and the deformed image, respectively; $f_{m}$ and $g_{m}$ represent the average gray level of the subset of the reference image and the deformed image, respectively; $\vec{p}$ represents six deformation parameters (u, v, $u_{x}$, $u_{y}$, $v_{x}$, and $v_{y}$), which represent the displacement of the center point of the subset and the four partial derivatives of the displacement and determine the shape function of the corresponding point coordinate between the reference image and the deformation image. After Newton–Raphson iteration, high-precision sub-pixel matching between points from the reference image to the deformed image can be realized.

**Figure 3:**
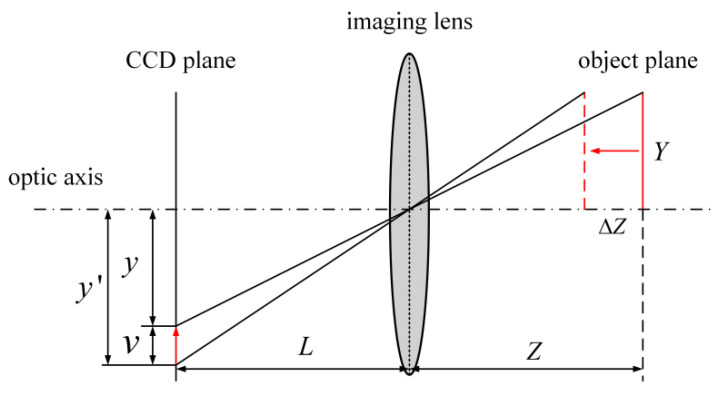
Effect of out-of-plane displacement on in-plane displacement.

In contexts in which both in-plane and out-of-plane displacements occur simultaneously, utilizing 2D-DIC to determine the in-plane displacement may lead to a certain degree of deviation. As such, the implementation of supplementary apparatus is necessary to rectify such discrepancies. Generally, the linearity between image displacement as computed by 2D-DIC and the corresponding physical displacement is considered axiomatic:

(5) $$\left\{\begin{array}{l} u=MU\\ v=MV \end{array}\right.$$

where M=L/Z is called the magnification factor; L is the image distance; and Z is the object distance. However, when the object has out-of-plane displacement, the in-plane displacement will be changed, as shown in Figure 3. Assume that the coordinates of the object are $\left(X,Y\right)$:

(6) $$\left\{\begin{array}{l} u\left(\Delta Z\right)\approx \frac{L}{Z}X\frac{\Delta Z}{Z}\\ v\left(\Delta Z\right)\approx \frac{L}{Z}Y\frac{\Delta Z}{Z}\\ \varepsilon_{xx}=\varepsilon_{yy}\approx -\frac{\Delta Z}{Z} \end{array}\right.$$

Based on Figure 3, it can be observed that alterations to the out-of-plane displacement *Z* will lead to changes in the in-plane displacement *y* of the object with height on the CCD plane, resulting in *y*′. As indicated by Formula (6), an object in close proximity to the lens will generate biaxial tensile virtual strain, with the magnitude increasing proportionally to ΔZ (the out-of-plane displacement). A non-uniform ΔZ will consequently cause non-uniform virtual strain. To achieve a closer approximation of the actual in-plane displacement *y*, compensation of the in-plane displacement can be achieved through increasing the object distance or utilizing a telecentric lens as opposed to a standard lens.

**Figure 4:**
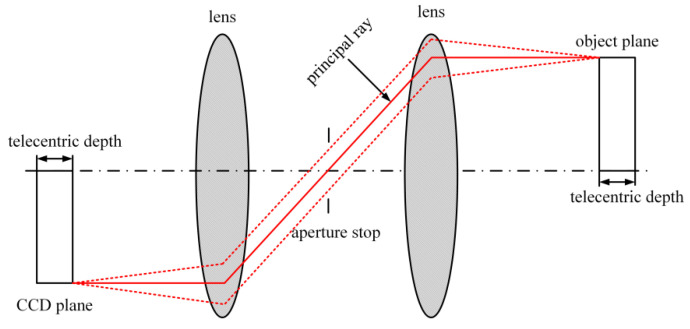
Optical path of telecentric lens.

The optical path diagrams of telecentric lenses are presented in Figure 4. It can be inferred that such lenses exhibit insensitivity to alterations in both the object distance and image distance within the telecentric depth. As such, objects imaged within the telecentric depth are capable of displaying highly consistent magnification values. The resultant consistency in image quality significantly enhances the accuracy of displacement and strain measurements in DIC analysis.

## 3. Experiment

### 3.1. Measurement of System Accuracy

**Figure 5:**
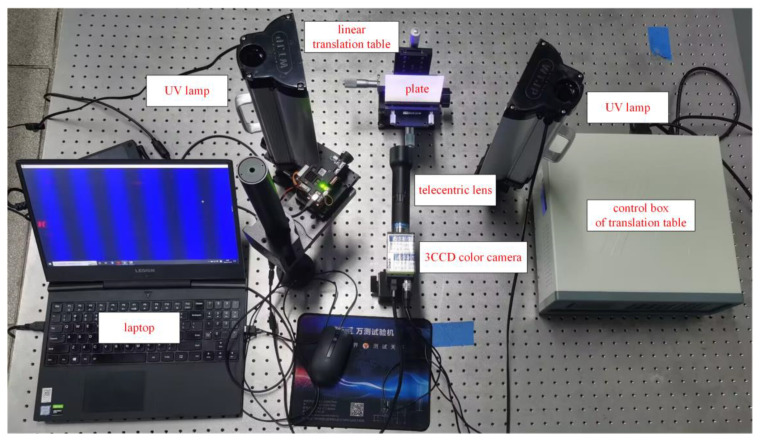
Experimental device diagram.

To quantitatively assess the measurement accuracy of the system, a rigid body translation experiment was conducted on a standard plate featuring red fluorescent speckles on its surface. The experimental setup, as depicted in Figure 5, involved the use of a DLP projector (brand: DLP3010; model: OPR305185) for projecting a computer-generated blue fringe pattern onto the object being measured. A 3CCD color camera (brand: JAI; model: AP-1600T-PGE) equipped with a telecentric lens (brand: Edmund; model: Edmund63074; magnification: 0.5) was employed to capture the patterns before and after deformation. The standard flat plate, measuring 100 × 100 mm × 1 mm and made from acrylic material, was securely clamped onto a linear translation platform (brand: Zolix; model: SK25A-65C) for the purposes of the experiment. The translation platform possessed the capacity to translate rigid bodies along the *x*, *y*, and *z* axes, which could be manipulated via the control box. The translation module boasted a high minimum resolution of 0.01 mm. An arrangement of two UV lamps, specifically UV curing lamps (brand name: WTJP, model number: undisclosed), each possessing an output power of 40 W and a wavelength of 365 nm, were symmetrically placed on either side of the specimen being tested. This configuration enabled the camera’s CCD target to receive light emitted by the red fluorescent speckles.

**Figure 6:**
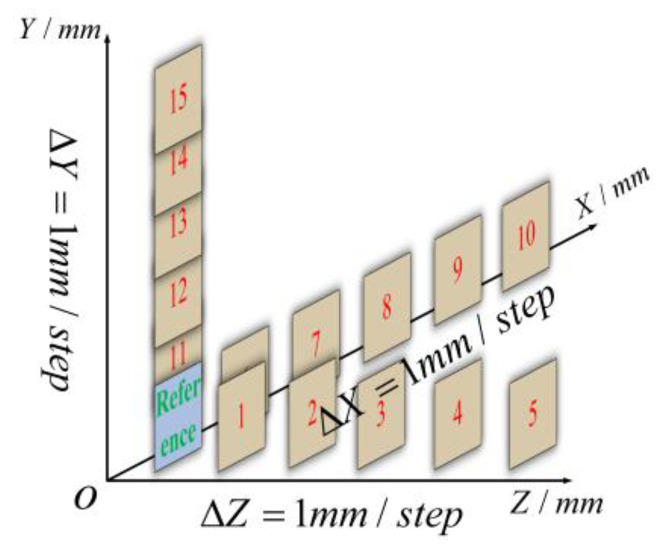
Measuring positions of standard flat plate.

**Figure 7:**
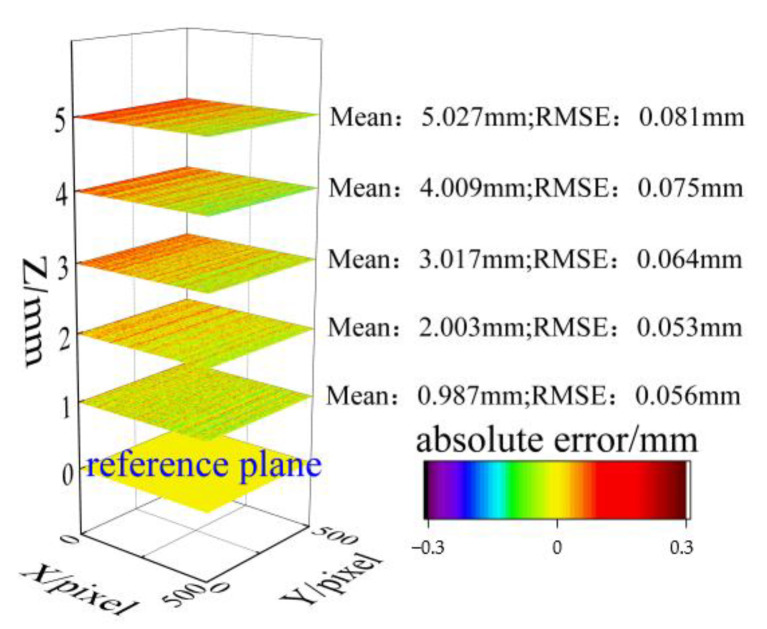
Accuracy evaluation of out-of-plane displacement.

**Figure 8:**
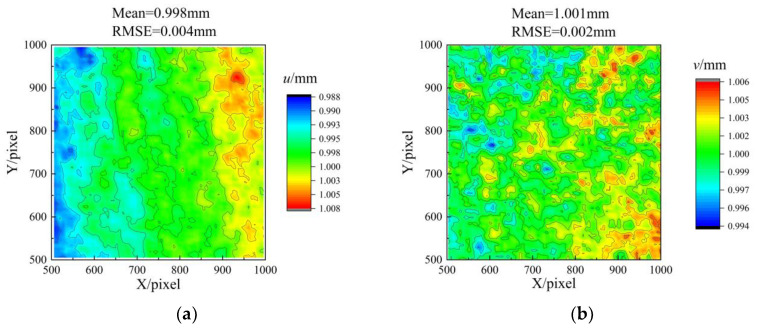
Accuracy evaluation of in-plane displacements. (**a**) Displacement *u* distribution at position 6, (**b**) Displacement *v* distribution at position 11.

In accordance with the experimental procedure presented in Figure 6, the flat plate was translated along the three orthogonal directions of *x*, *y*, and *z*, with each translation step set to 1 mm. Meanwhile, the numerical labels ‘1–15’ simultaneously serve as indicators of both position and sequence. Figure 7 displays the absolute error distribution of the entire measuring range for the five positions, where the RMSE ranged from 0.053 mm to 0.081 mm. Based on the results presented in Table 1, it was evident that the maximum MAE was 0.027 mm, while the minimum MAE was 0.003 mm. The displacement distributions at positions 6 and 11 are depicted in Figure 8a,b, respectively. It was noteworthy that the comparison of the in-plane and out-of-plane displacement measurement indicated a significantly lower RMSE for the in-plane displacement. Additionally, the measurement of direction *y* demonstrated a minor superiority over that of direction *x*.

**Figure 9:**
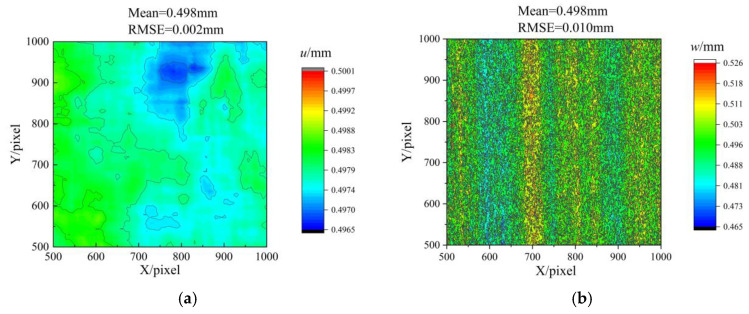
Measurement results when moving 0.5 mm simultaneously in the *x* and *z* directions. (**a**) Measurement results of displacement u, (**b**) Measurement results of displacement w.

The displacement measurement results for simultaneous movements of 0.5 mm in the *x*, *y*, and *z* directions are presented in Figure 9. The data indicated a comparatively small error in displacement *u* relative to displacement *w*. Notably, the use of a telecentric lens precluded the occurrence of biaxial tensile effects in the displacement *u* distribution. Consequently, measurements of the in-plane displacement remained unaffected by the out-of-plane displacement.

A conclusion was drawn based on the rigid translation experiment that the method proposed in this article can be used for 3D deformation measurement, and there was almost no crosstalk between the in-plane deformation and out-of-plane deformation.

### 3.2. Free-End-Loading Experiment with Cantilever Beam

**Figure 10:**
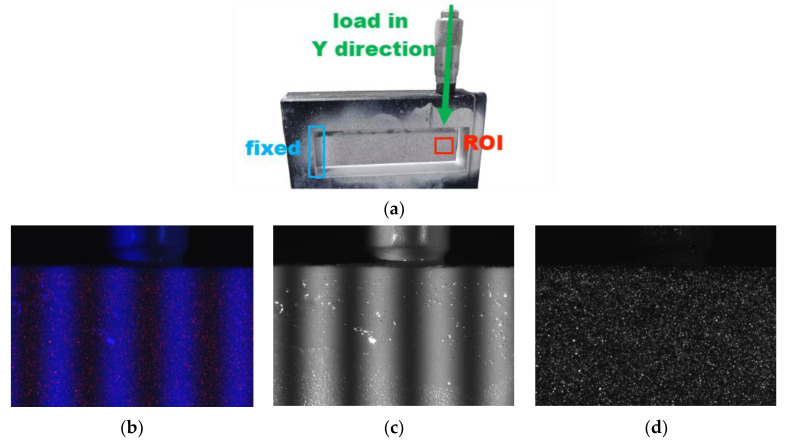
Captured image of a cantilever beam. (**a**) Schematic diagram of cantilever beam, (**b**) Color image, (**c**) Blue channel image, (**d**) Red channel image.

An in-plane loading experiment was performed on a cantilever beam with the cantilever beam specimen depicted in Figure 10. The left end of the cantilever beam was fixed, and a known displacement was applied in the y direction at the free end of the specimen. The measured Region of Interest (ROI) corresponded to the red box illustrated in Figure 10a. The camera utilized in the experiment possessed a 2064 × 1544 pixel resolution and a physical size of approximately 14 mm × 10 mm. The experiment captured a color image, blue channel image, and red channel image, which are presented in Figure 10b–d.

**Figure 11:**
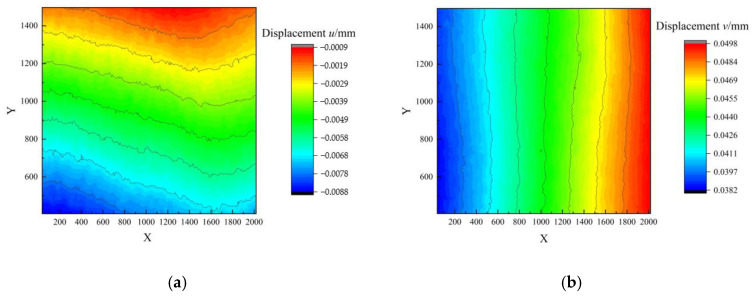
Measurement results when the in-plane load was 0.05 mm. (**a**) Measurement results of displacement u, (**b**) Measurement results of displacement v.

Following the application of a known displacement of 0.05 mm in the y direction at the free end of the cantilever beam, the associated displacement field was meticulously measured. Specifically, Figure 11 manifests the quantified displacements u and v, which are presented in (a) and (b), respectively. The experimental results agreed well with the theoretical displacement distribution.

**Figure 12:**
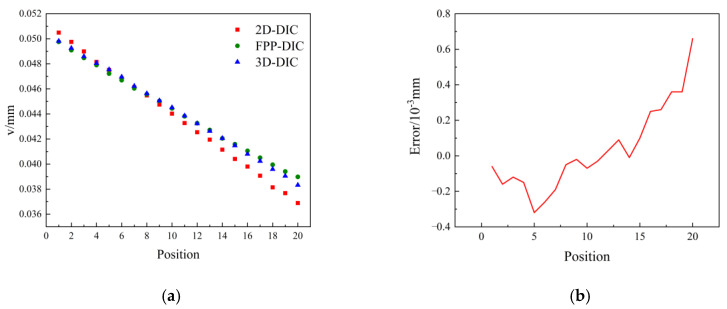
Comparison with the traditional methods in the middle row of a cantilever beam. (**a**) Measurement value, (**b**) Error.

In this study, a comparison was made between the measured results obtained using the 2D-DIC method and the 3D-DIC method, as depicted in Figure 12a. Both the 2D-DIC and 3D-DIC methods employed the same black-and-white CCD camera (brand: Basler; model: acA4112-30; resolution: 4096 × 3008 pixels) and 35 mm Kowa low-distortion fixed-focus lens. A set of 20 measurement points situated in the central area of the cantilever beam were extracted and compared, as shown in Figure 12b, revealing that the maximum error between the FPP-DIC and 3D-DIC methods was 0.0007 mm. However, since there existed an out-of-plane displacement in the experiment and the 2D-DIC did not adopt a telecentric lens, the measurement results for the 2D-DIC exhibited a certain deviation from those obtained using the 3D-DIC and FPP-DIC methods.

A conclusion was drawn based on the in-plane loading experiment with the cantilever beam that the method proposed in this article had a high accuracy in measuring in-plane displacement and that the use of a telecentric lens avoided the influence of the out-of-plane displacement on the in-plane displacement measurement.

### 3.3. Center-Loading Experiment of Disk

**Figure 13:**
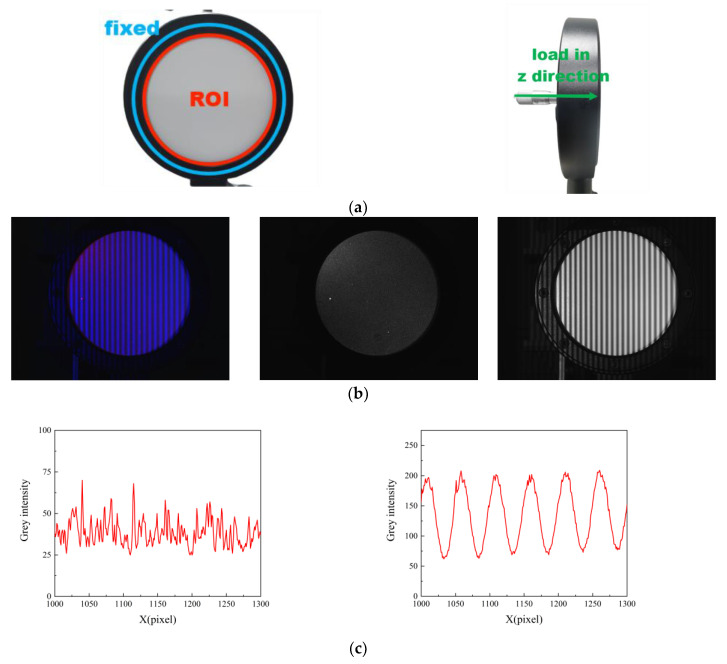

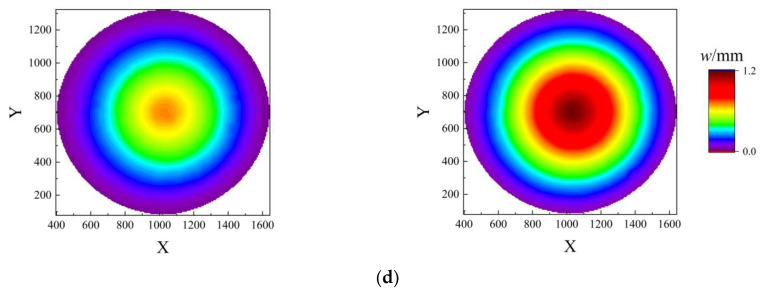
Measurement results of disk center loading. (**a**) Schematic diagram of disk, (**b**) Color image, red channel image, and blue channel image, (**c**) Gray distribution of red channel image and blue channel image, (**d**) Measurement results for displacement *w* (deformed image 1 and deformed image 2 from left to right).

**Figure 14:**
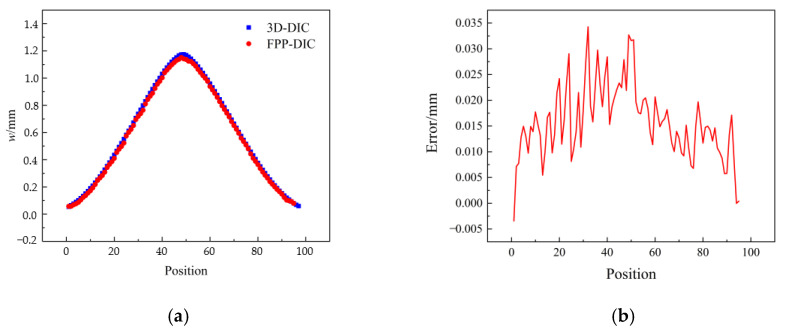
Comparison with 3D-DIC in the middle row of the disk. (**a**) Measurement value, (**b**) Error.

The 3D deformation of the center-loaded disk was evaluated in this study. The disk, as depicted in Figure 13a, was secured in place while the loading device, which applied a load along the *z*-axis, was installed at its center. Color images, red channel images, and blue channel images are presented in Figure 13b. Figure 13c shows the gray distribution of the red channel image and the blue channel image. It can be seen that the gray distribution of the speckle pattern in the red channel was disorderly, while the gray distribution of the fringe pattern in the blue channel was sinusoidal, which was consistent with the theoretical distribution trend. This can explain why there was almost no crosstalk between the speckle pattern and the fringe pattern. The displacement *w* was measured, and the results obtained by extracting 100 equally spaced measurement data points in the center row are depicted in Figure 14. A comparison between the measured results and those obtained from the 3D-DIC technique revealed a maximum error of 0.034 mm for the FPP-DIC method. In Figure 14b, it can be seen that the maximum error occurred at positions 30 and 50. The possible reason is that the slope was relatively large at position 30, while position 50 was a peak value. At these special positions, the measurement results for 3D-DIC and FPP-DIC may not match well.

A conclusion was drawn based on the center-loading experiment with the disk that the method proposed in this article can effectively measure the out-of-plane displacement.

## 4. Conclusions

In this paper, we proposed a novel approach that combined the FPP technique with fluorescence DIC to effectively and accurately measure 3D shape and deformation simultaneously. Specifically, we leveraged the utilization of a 3CCD color camera to efficiently distinguish and acquire information from both blue light fringe patterns and red fluorescent speckle patterns. The FPP method and DIC method were applied to effectively measure the out-of-plane displacement and in-plane displacement, respectively, while a telecentric lens was introduced to precisely correct the in-plane displacement. The error of this method was quantified to be lower than 0.027 mm, as verified through rigorous rigid body translation experiments. Finally, we empirically demonstrated the efficacy and versatility of our proposed approach via free-end-loading tests on a cantilever beam as well as a center-loading test on a disk.

Notwithstanding the utility of the phase-shifting method for static measurement, its applicability is restricted in circumstances where dynamic measurement is necessitated. To this end, alternative signal-processing techniques such as Fourier Transform and Wavelet Transform may be employed. Likewise, the use of binary projection technology holds promise for accelerating projection speed and thus is an area warranting future research.

## Figures and Tables

**Table 1:** Measurement errors of out-of-plane displacements.

*W*/mm	MAE/mm	RMSE/mm	Error/%
1	0.013	0.056	1.3
2	0.003	0.053	0.15
3	0.017	0.064	0.57
4	0.009	0.075	0.23
5	0.027	0.081	0.54

## Data Availability

Data sharing not applicable.
